# Stretchable Ag/AgCl Nanowire Dry Electrodes for High-Quality Multimodal Bioelectronic Sensing

**DOI:** 10.3390/s24206670

**Published:** 2024-10-16

**Authors:** Tianyu Wang, Shanshan Yao, Li-Hua Shao, Yong Zhu

**Affiliations:** 1 National Key Laboratory of Strength and Structural Integrity, Institute of Solid Mechanics, School of Aeronautic Science and Engineering, Beihang University, Beijing 100191, China; by2305116@buaa.edu.cn; 2 Department of Mechanical Engineering, Stony Brook University, Stony Brook, NY 11794, USA; shanshan.yao@stonybrook.edu; 3 Department of Mechanical and Aerospace Engineering, North Carolina State University, Raleigh, NC 27695, USA; yzhu7@ncsu.edu

**Keywords:** bioelectrical signal measurement, dry electrode, electrocardiogram, electroencephalogram, electromyogram

## Abstract

Bioelectrical signal measurements play a crucial role in clinical diagnosis and continuous health monitoring. Conventional wet electrodes, however, present limitations as they are conductive gel for skin irritation and/or have inflexibility. Here, we developed a cost-effective and user-friendly stretchable dry electrode constructed with a flexible network of Ag/AgCl nanowires embedded in polydimethylsiloxane (PDMS). We compared the performance of the stretched Ag/AgCl nanowire electrode with commonly used commercial wet electrodes to measure electrocardiogram (ECG), electromyogram (EMG), and electroencephalogram (EEG) signals. All the signal-to-noise ratios (SNRs) of the as-fabricated or stretched (50% tensile strain) Ag/AgCl nanowire electrodes are higher than that measured by commercial wet electrodes as well as other dry electrodes. The evaluation of ECG signal quality through waveform segmentation, the signal quality index (SQI), and heart rate variability (HRV) reveal that both the as-fabricated and stretched Ag/AgCl nanowire electrode produce high-quality signals similar to those obtained from commercial wet electrodes. The stretchable electrode exhibits high sensitivity and dependability in measuring EMG and EEG data, successfully capturing EMG signals associated with muscle activity and clearly recording *α*-waves in EEG signals during eye closure. Our stretchable dry electrode shows enhanced comfort, high sensitivity, and convenience for curved surface biosignal monitoring in clinical contexts.

## 1. Introduction

In modern medical diagnosis and treatment, monitoring various bioelectrical signals has become an important task. Among these bioelectrical signals, electrocardiogram (ECG), electromyogram (EMG), and electroencephalogram (EEG) are the most common types. The ECG signal represents the electrophysiological activity of the cardiomyocytes of the human heart, containing rich information on cardiac activity. ECG has been widely used in the clinical diagnosis of various cardiovascular diseases and the real-time monitoring of cardiac function [1,2]. Monitoring EMG signals related to muscle activity can capture gestures and achieve real-time control of robotic hands [3,4]. The accurate monitoring of EEG signals can deepen our understanding of increasingly attention-grabbing diseases such as dementia and depression [5,6,7]. Among these signals, action potentials of brain neural activity are the smallest in the human body and are significantly affected by noise [8].

Monitoring these signals requires the use of electrodes. Traditional electrodes are wet silver/silver chloride (Ag/AgCl) electrodes, which have been widely used clinically. However, these wet electrodes are generally pre-gelled and disposable, meaning that they need to be fixed on the skin using conductive gels [9,10]. As a result, two problems arise. The first is discomfort and risk of allergies (severe problems such as herpes and ulcers can result) [11,12]. The second is that the conductive gel can dehydrate and harden, causing the failure of the wet electrode [13,14]. Sometimes, electrolyte liquids are added multiple times to extend their service life, but the stability and reliability of the electrode can decrease during this process [15,16]. Therefore, traditional wet electrodes are not suitable for the long-term monitoring of bioelectrical signals. In modern medical diagnosis and treatment, monitoring bioelectrical signals for a long time is often required [17,18], which puts forward requirements for the comfort and service life of electrodes.

To address the issues associated with wet electrodes, researchers have recently started exploring the use of novel electrode materials in so-called dry electrodes (gel-free) [4,18,19,20,21,22,23,24,25,26,27,28,29,30,31,32,33,34,35], including metal plate electrodes [19], semi-dry electrodes [20,36], fabric electrodes [21,22], microneedle electrodes [23], and porous electrodes [4]. However, existing dry electrodes often suffer from higher signal noise and poorer signal stability [19]. To overcome these issues, researchers have attempted various flexible dry electrode designs and preparation methods, including the use of materials such as carbon nanotubes [28,33], metal nanowires [24,25,26], capacitive epidermal electronic system designs [37], metal thin films [38], and conductive polymers [27]. In particular, silver nanowires (Ag NWs) have been studied as an excellent electrode material with high conductivity, sensitivity, and signal stability [39,40,41,42,43,44,45]. Myers et al. developed a dry electrode based on Ag NWs for non-invasive and wearable ECG sensing and compared the collected ECG signals with those collected using traditional wet Ag/AgCl electrodes [39]. The Ag NW electrode was able to collect acceptable ECG signals, but some baseline drift and distortion were observed [24]. This represents the main disadvantage of dry electrodes: a low signal-to-noise ratio. Porous electrodes were found capable of measuring comparable signals with or without deformation, including stretching and bending, etc. However, the signals were less stable and of relatively poor quality, which is challenging, especially when measuring EEG signals. Ag NW electrodes combine flexibility with high signal quality but still cannot match the signal-to-noise ratio obtained by wet electrodes. It remains challenging to dramatically improve the signal quality (to be comparable to wet Ag/AgCl electrodes) while maintaining the flexibility of the dry electrode.

In this work, we utilized electrochemical methods to fabricate a novel flexible dry electrode composed of Ag/AgCl NW. Using this electrode, we monitored a variety of bioelectrical signals, including ECG, EMG, and EEG, and compared the results with those obtained using commercially available wet Ag/AgCl electrodes from 3M. Our dry electrode produced high-quality ECG, EMG, and EEG signals and demonstrated high sensitivity, low noise, high stability, and excellent reproducibility. Of note is that it was able to measure EEG signals that were previously difficult to detect effectively using dry electrodes. The signal-to-noise ratios of the ECG, EMG, and EEG signals were comparable to those of commercial wet electrodes. Additionally, due to its flexibility, our dry electrode was capable of measuring excellent signals even under 50% elongation, making it more suitable for wearable applications where skin deformation is expected. This simple and cost-effective manufacturing process makes our new Ag/AgCl NW flexible dry electrode promising for clinical trials and commercial production.

## 2. Materials and Methods

### 2.1. Electrode Preparation

**Figure 1:**
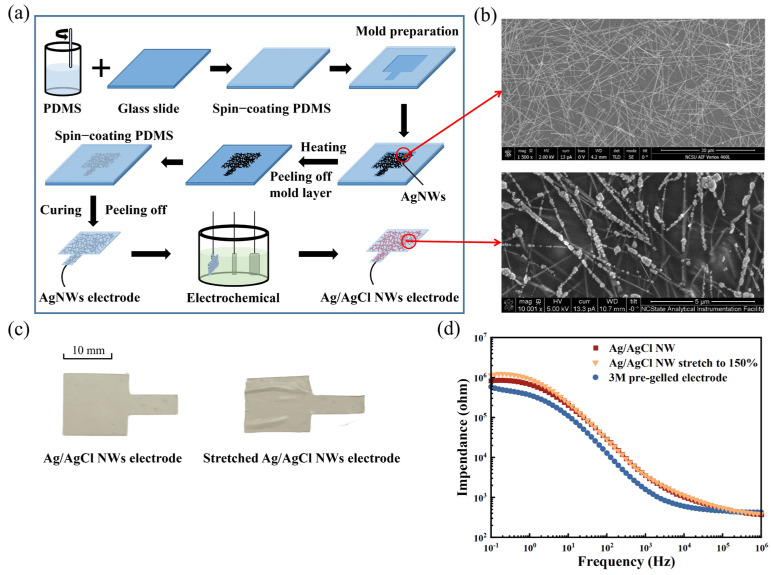
(**a**) Preparation of Ag/AgCl NW dry electrode, (**b**) scanning electron micrographs of Ag NW electrodes and Ag/AgCl NW electrodes, (**c**) photographs of Ag/AgCl NW electrode before and after tensile deformation, and (**d**) impedance of each electrode.

Figure 1a shows the fabrication process of Ag/AgCl NW electrodes. The polydimethylsiloxane (PDMS) pre-polymer solution was prepared by mixing the PDMS pre-polymer and crosslinker with a mass ratio of 10:1 (Sylgard 184, Dow Corning, Midland, MI, USA). Firstly, the PDMS solution was spin-coated on a glass slide and then cured at 80 °C for two hours to make the mold as well as the final PDMS layer of the electrode. The synthesis of Ag NWs was achieved via the polyol method; then, they were dispersed in ethanol to a concentration of 0.02 mg/mL. The length of the Ag NW was controlled to be 20–60 μm with an average diameter of 90 nm. This diluted Ag NW suspension was then dropped onto a glass slide with a PDMS mold through drop-casting. In the meantime, the glass slide was put on a hot plate with a temperature of 50 °C to facilitate the evaporation of the solvent, resulting in the formation of a random network of Ag NWs. After peeling off the mold layer and curing the spin-coated thin PDMS layer, the Ag NW/PDMS electrode was peeled off the glass slide with the Ag NW network on top of the surface [25]. For electrochemical deposition, the Ag NW electrode was connected to the potentiostat using a three-electrode system, where the Ag NW electrode served as the working electrode with a standard Ag/AgCl electrode as the reference electrode, and a platinum plate as the counter electrode. A NaCl solution (1 mol/L) was selected as the electrolyte solution, and a voltage of 1.7 V was applied until the current stabilized to produce a Ag/AgCl NW electrode. Figure 1b shows the scanning electron microscopy images of the Ag NW electrode as well as the Ag/AgCl NW electrode, respectively. After electrochemical treatment, a large number of bulk AgCl components (e.g., particles) are attached to the Ag NW network, constituting the Ag/AgCl NW network. Figure 1c illustrates a Ag/AgCl NW electrode before and after it was stretched. After being subjected to a considerable degree of stretching, the Ag/AgCl NW electrodes showed no apparent damage. As shown in Figure 1d, the impedances of the Ag/AgCl NW electrode and the stretched 150% strain Ag/AgCl NW electrode were slightly higher than that of the commercial wet electrode but within the same order of magnitude.

Regarding the biocompatibility of dry electrodes, all materials used in this work, namely AgNWs, AgCl particles, and PDMS, have shown good biocompatibility in various studies in the past. Ag/AgCl is often used by coming into contact with human skin in commercial wet electrodes in the clinic, and both AgNWs and AgCl particles have been shown to have good skin friendliness, antimicrobial properties, and low toxicity [26,46,47]. PDMS has been frequently used as a substrate for a wide range of bio-compatible membranes in biological tests; it has good skin-friendliness and has even been used to produce wound dressings [26,48]. In all experiments here, there was no allergy or discomfort during the prolonged test based on the good biocompatibility of the Ag/AgCl NW electrode and PDMS.

### 2.2. Experimental Methods

The PowerLab 26T (an ADI instrument, Norwood, MA, USA) was used to measure ECG, EMG, and EEG signals from both Ag/AgCl NW electrodes and commercial 3M Ag/AgCl pre-gelled wet electrodes. The data were displayed and recorded using LabChart 7 software at a sampling frequency of 1000 Hz. Note that skin was untreated during measurements, and raw signals were collected without any filtering. The Ag/AgCl NW electrodes were secured to the skin using medical tapes.

**Figure 2:**
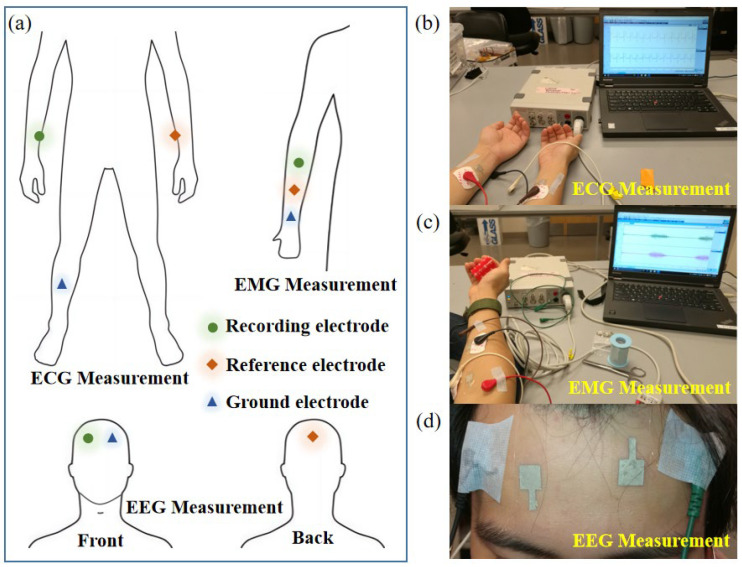
Multiple bioelectrical signal measurement methods: (**a**) electrode arrangement for different bioelectric signal measurements. Experimental setup and measurement site: (**b**) ECG measurement, (**c**) EMG measurement, and (**d**) EEG measurement.

Figure 2 shows the electrode placements for the ECG, EMG, and EEG measurements. For all measurements, the Ag/AgCl NW electrode and the commercial wet electrode were placed in adjacent positions. Due to the adhesive nature of PDMS, the Ag/AgCl NW electrode can be applied directly to the skin without other fixation measures. As the subject perspires, the Ag/AgCl NW electrode adheres more closely to the subject’s skin. During the experiment, the subject remained stationary. For ECG measurements, the recording and reference electrodes were attached to the left and right wrists, respectively, and the ground electrodes were attached to the right leg according to Einthoven’s triangle [49]. For EMG measurements, the recording electrode was placed in the internal elbow socket, the reference electrode was located in the middle of the forearm, and the ground electrode was located close to the wrist. During the experiment, the objects were gripped with different forces [50]. For EEG measurements, the recording and reference electrodes were placed on the forehead and the occipital lobe on the scalp at the back of the head, respectively, and the electrode at the forehead for grounding [16]. During the experiment, the subject remained calm with their eyes closed, then opened their eyes and moved their eyeballs several times.

### 2.3. Signal Evaluation Methods

#### 2.3.1. ECG Signal Evaluation Method

The standard ECG signal exhibits five types of standard waveforms [17] (P, QRS complex, and T waveforms) in each cycle, which can be visually identified. Therefore, upon obtaining the ECG signal, an automatic algorithm can be used to identify and classify these five waveforms as a preliminary test for signal quality. In this study, the open-source Python toolkit neurokit2 [51] was used for waveform recognition and classification. During the preliminary visual inspection, the signal was not post-processed in order to reveal the characteristics of the original signal.

SQI parameters are often used as a reference to assess the quality of ECG signals [52]. In order to ensure the accuracy of these parameters, it is necessary to process the raw signal by eliminating noise and improving the accuracy of peak detection. In this study, the method proposed by Elgendi et al. [53] was used for raw signal processing.

Different SQI parameters have varying degrees of importance and evaluation criteria. Calculating too many SQI parameters is not only costly but also fails to consider the coupling relationships between each parameter, leading to the inaccurate assessment of ECG signal quality. To better assess the quality of the obtained ECG signal, this study employed a fuzzy comprehensive evaluation method proposed by Zhao et al. [54]. This method first calculates the following three SQI parameters:

kSQI, the fourth moment of the ECG signal, can be used as an independent indicator of signal quality. When kSQI is greater than 5, the signal is considered to have excellent quality [55].

pSQI, which represents the relative power in the QRS complex, can also be used as an independent indicator of signal quality. When pSQI falls within the range of 0.5 to 0.8, the signal is considered to have excellent quality [55].

(1) $$\mathrm{pSQI}=\frac{\int_{5\mathrm{Hz}}^{15\mathrm{Hz}}P\left(f\right)d\;f}{\int_{5\mathrm{Hz}}^{40\mathrm{Hz}}P\left(f\right)d\;f}$$

basSQI, which represents the relative power in the baseline, can be used as another independent indicator of signal quality. It is considered that the closer basSQI is to 1, the higher the quality of the signal will be [56].

(2) $$\mathrm{basSQI}=1-\frac{\int_{0\mathrm{Hz}}^{1\mathrm{Hz}}P\left(f\right)d\;f}{\int_{0\mathrm{Hz}}^{40\mathrm{Hz}}P\left(f\right)d\;f}$$

Afterward, a fuzzy comprehensive evaluation method combining Cauchy distribution, rectangular distribution, and trapezoidal distribution was employed to calculate the membership degree of the signal to be evaluated based on the optimal logical combination of the parameters mentioned above for the best accuracy. By establishing a fuzzy vector and selecting a bounded computational sub-fuzzy synthesis, a vector of fuzzy comprehensive evaluation was obtained. Then, the final evaluation parameter *v* was obtained through the principle of weighted membership:

(3) $$v=\frac{\sum_{j=1}^{3}s_{j}^{2}\cdot j}{\sum_{j=1}^{3}s_{j}^{2}}$$

where *s* is the fuzzy vector resulting from processing the three SQI parameters by the fuzzy synthesis algorithm. *s_j_* represents the *j*th element in the fuzzy vector *s*. The final evaluation grade set is as follows:

(4) $$\mathrm{ECG}\;\left\{\begin{array}{l} \mathrm{Excellent}\;(E),\;v\leq 1.50;\\ \mathrm{Barely}\;\mathrm{acceptable}\;(B),\;1.50<v<2.40;\\ \mathrm{Unacceptable}\;(U),\;v\geq 2.40. \end{array}\right.$$

Thus, for any ECG signal to be evaluated, a quality assessment mechanism based on fuzzy comprehensive evaluation can be used to obtain the ECG quality assessment results according to the above formula:

If it is E, the ECG signal quality is good. The signal can be directly input into identification, safety monitoring, or other applications.

If it is U, three SQIs can be analyzed: if kSQI or basSQI does not qualify, there is noise and artifacts. First, denoise the signal and then re-evaluate the quality of the electrocardiogram. If pSQI is not qualified, the electrocardiogram of the tester needs to be re-recorded.

If it is B, the ECG quality assessment should be performed again. If the result is E, the signal should be processed according to Method 1. Otherwise, process it according to Method 2.

Heart rate variability (HRV) refers to the change in successive heartbeats, which contains information about the regulation of the cardiovascular system by neurohumoral factors. It may be a valuable indicator for predicting cardiac arrest and arrhythmia events and evaluating the severity and prevention of cardiovascular diseases [2]. Therefore, for subjects with normal cardiac indicators, calculating and analyzing HRV indices obtained from ECG signals can also determine the quality and reliability of the signal. Generally, there are two types of HRV parameters: time domain and frequency domain. In this study, the following HRV parameters were selected for calculation and analysis.

In the time domain, the following can be found:

Mean-NN: the average of R-peak to R-peak (NN) intervals between adjacent sinus heartbeats recorded in the signal.
ECG Rate Mean: the average heart rate in the ECG signal.
RMSSD: the root mean square of the differences between adjacent NN intervals.
SDNN: the standard deviation of the differences between adjacent NN intervals.
PNN50: the percentage of consecutive NN intervals that differ by more than 50 ms.
SD1: the length of the minor axis of the ellipse fitted to the Poincare Plot of NN intervals.

In the frequency domain, the following can be found:

VLF: the power in the frequency band between 0 and 0.04Hz.
LF: the power in the frequency band between 0.04 Hz and 0.15 Hz.
HF: the power in the frequency band between 0.15 Hz and 0.4 Hz.
VHF: the power in the frequency band greater than 0.4 Hz.

Generally, high-quality ECG signals have most of their HRV power in the frequency band between 0.04 Hz and 0.4 Hz after being subjected to a fast Fourier transform (FFT) of the entire ECG signal [57]. Therefore, observing the distribution of energy in the frequency domain can determine the quality and reliability of the signal.

#### 2.3.2. EMG Signal Evaluation Method

The evaluation of EMG signals is relatively straightforward. When muscle activity is activated (in this study, the hand grip action activates forearm muscle activity), an EMG signal is generated. Different grip strengths result in different EMG signal amplitudes.

#### 2.3.3. EEG Signal Evaluation Method

EEG is generated by slow changes in the membrane potential of cortical neurons, especially excitatory and inhibitory postsynaptic potentials (EPSPs and IPSPs). An EEG waveform consists of component waves of different frequencies. These waves can be extracted to provide information about different brain activities. The α-wave, mainly ranging from 8 Hz to 13 Hz, is the predominant wave observed in awake adults. It is generally observable when the subject closes their eyes. The α-wave disappears when the subject opens their eyes or engages in mental work [4] (such as calculation or focusing on a thought). Therefore, monitoring changes in the EEG signal before and after the subject opens his or her eyes and during eye movement can help to assess the quality and sensitivity of the EEG signal.

The analysis of EEG signals is usually carried out in the frequency domain. This requires that the EEG signal measured in the time domain be converted to the frequency domain using a fast Fourier transform (FFT) to derive the power spectral density (PSD) versus frequency. PSD is a tool used to characterize the energy distribution of a signal at different frequencies. It represents the power or energy density of the signal at different frequencies. PSD is calculated by the following equation:

(5) $$\mathrm{PSD}\left(f\right)=\left(\frac{1}{N}\right){\left|X\left(f\right)\right|}^{2}$$

where *N* is the length of the signal (that is, the number of points at which the signal is sampled) and *X*(*f*) is the complex amplitude value of the signal in the frequency domain obtained from the fast Fourier transform.

Signal correlation is used to assess the consistency and similarity between two signals. The cross-correlation coefficient *ρ* between signal *X* and signal *Y* (two segments of the same length *L*) is defined as follows:

(6) $$\rho =\frac{\sum_{i=1}^{L}X_{i}\cdot Y_{i}}{\sqrt{\sum_{i=1}^{L}X_{i}^{2}\cdot \sum_{i=1}^{L}Y_{i}^{2}}}$$

where *ρ* is a number between 0 and 1. The larger *ρ* is, the stronger the correlation between the two signals. The signal correlation evaluation can be used to characterize the similarity of the signals measured by Ag/AgCl NW electrodes to those measured by established commercial wet electrodes. In this way, the reliability of the Ag/AgCl NW electrode is demonstrated.

The raw signals were subjected to band-pass filtering within specific frequency ranges: 0.3 to 100 Hz for ECG signals, 10 to 2000 Hz for EMG signals, and 0.3 to 50 Hz for EEG signals. Additionally, a 60 Hz notch filter was employed across all signals to eliminate power line interference. The signal-to-noise ratio (SNR) is defined as follows:

(7) $$\mathrm{SNR}=10\log_{10}\left(\frac{A_{\mathrm{signal}}^{2}}{A_{\mathrm{noise}}^{2}}\right)=20\log_{10}\left(\frac{A_{\mathrm{signal}}}{A_{\mathrm{noise}}}\right)$$

where *A*_signal_ and *A*_noise_ are the root mean square of the biopotential signals (ECG/EMG/EEG signals in this study) and the noise collected in the experiment, respectively. Generally, the SNR of the reported dry electrodes is smaller than that of wet electrodes, which is a common drawback of dry electrodes.

## 3. Results and Discussion

### 3.1. ECG Signal Measurement Results and Evaluation

**Figure 3:**
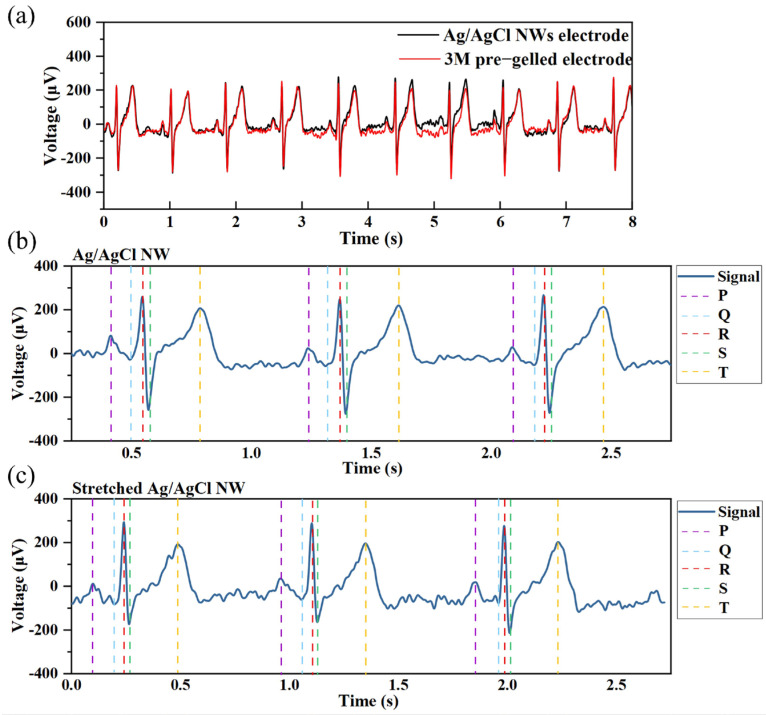
ECG signal: (**a**) part of the full-length raw signal measured by the Ag/AgCl NW electrode and commercial pre−gelled wet electrode, (**b**) the identification of the ECG signal waveform measured by the Ag/AgCl NW electrode, and (**c**) the identification of the ECG signal waveform measured by the Ag/AgCl NW electrode with a 50% tensile strain.

In Figure 3a, the raw signals measured by a commercial wet electrode and an Ag/AgCl NW electrode are presented. The waveforms of the two signals are similar, with a cross-correlation coefficient of 0.96, indicating a strong correlation. The SNRs of the signals measured by the commercial wet electrode and the Ag/AgCl NW electrode are 24.5 dB and 28.3 dB, respectively. It can be seen that the quality of the signals measured by our dry electrode reaches the level of or even exceeds that of the pre-gelled wet electrode. Figure 3b,c show ECG signals measured by the Ag/AgCl NW electrode without and with 50% strain, respectively. The dashed lines of different colors indicate the five typical waveforms of the ECG signal in one cycle. A clear P, QRS complex, and T waveforms can be observed. This indicates that the Ag/AgCl NW electrode can measure ECG signals with high quality both before and after stretching. The noise level of the signal measured by the stretched Ag/AgCl NW electrode is slightly higher than that measured by the original (as-fabricated) electrode. However, since the original ECG signal requires preprocessing before specific parameter analysis, a small amount of noise will not significantly affect the signal quality.

Table 1 presents the raw signals (full length) measured by the commercial wet electrodes, Ag/AgCl NW electrodes, and Ag/AgCl NW electrodes with 50% tensile strain, which were processed by Elgendi’s filtering algorithm to obtain three SQI parameters, and the signal quality was evaluated using the fuzzy algorithm proposed by Zhao et al. [54]. It can be seen that the kSQI, pSQI, and basSQI of the signals measured by the three electrodes all meet the requirements, indicating the high quality of the ECG signals. The *v* value (as defined in Equation (3)) of the three signals measured by the three electrodes using Zhao’s algorithm is 1.17, and according to Equation (4), the quality of these signals is “excellent”.

**Figure 4:**
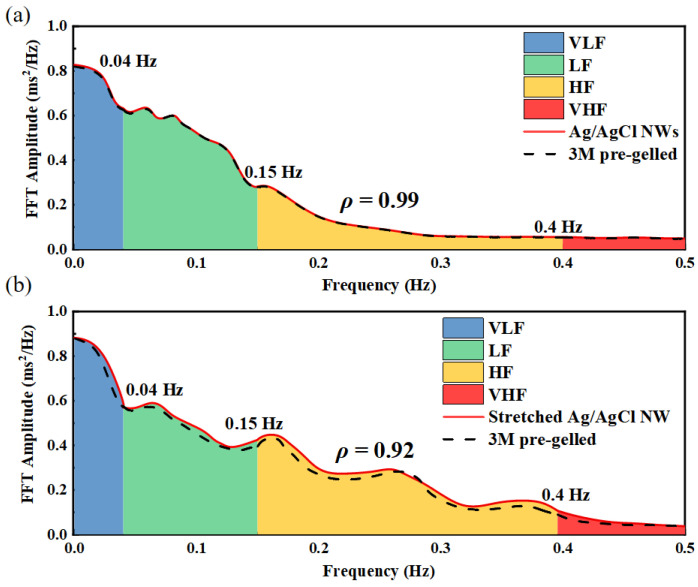
Average FFT amplitudes of NN sequences of full-length ECG signals measured at each electrode: (**a**) Ag/AgCl NW dry electrode with commercial wet electrode; (**b**) Ag/AgCl NW dry electrode at 50% tensile strain with commercial wet electrode.

As shown in Figure 4, the average FFT amplitude of the NN sequence of ECG signals (full length) measured by commercial pre-gelled wet electrodes, Ag/AgCl NW electrodes, and stretched Ag/AgCl NW electrodes are presented. The cross-correlation coefficient between the two electrodes in Figure 4a is 0.99, indicating an excellent match between the two electrodes, with most of the energy concentrated below 0.4 Hz. In Figure 4b, the ECG signal measured by the stretched Ag/AgCl NW electrode is more severely affected by baseline drift and motion artifacts, but the cross-correlation coefficient is still strong at 0.92, indicating a strong correlation between the signal measured by the two electrodes, with most of the energy also concentrated below 0.4 Hz. This demonstrates that the ECG signals measured by the Ag/AgCl NW electrodes are high quality, without or with stretching.

**Figure 5:**
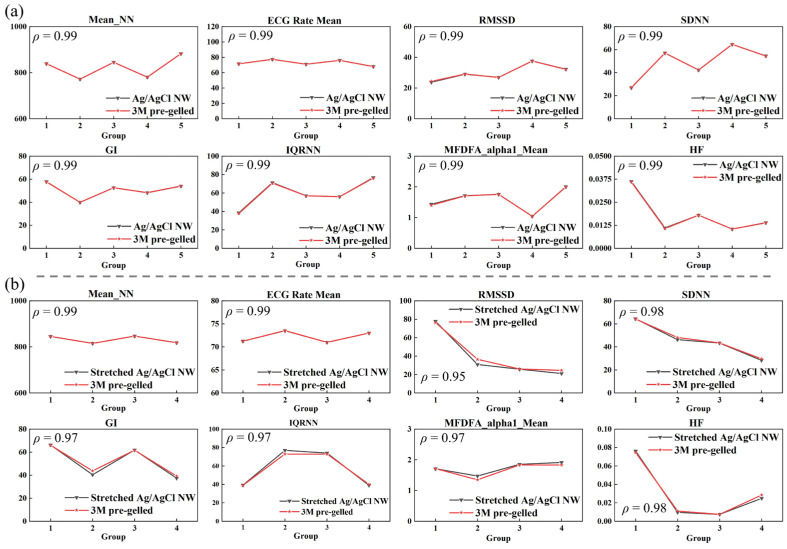
HRV parameters of ECG signals of random slices measured by each electrode: (**a**) Ag/AgCl NW dry electrode with commercial wet electrode, (**b**) Ag/AgCl NW dry electrode at 50% tensile strain, and commercial wet electrode.

In order to compare the signals measured with the Ag/AgCl NW electrodes and the commercial pre-gelled wet electrodes, the full-length signal was randomly divided into multiple groups, and HRV parameters were examined for each group. The summarized results are shown in Figure 5. Figure 5a shows that the HRV parameters from the Ag/AgCl NW electrodes are highly correlated with those from commercial wet electrodes. As shown in Figure 5b, although the correlation of the stretched Ag/AgCl NW electrode is slightly lower than that of the unstretched one, they still exhibit a strong correlation. This demonstrates that Ag/AgCl NW electrodes have good stability and reliability and can be used as ECG signal acquisition electrodes in clinical settings.

### 3.2. EMG Signal Measurement Results and Evaluation

**Figure 6:**
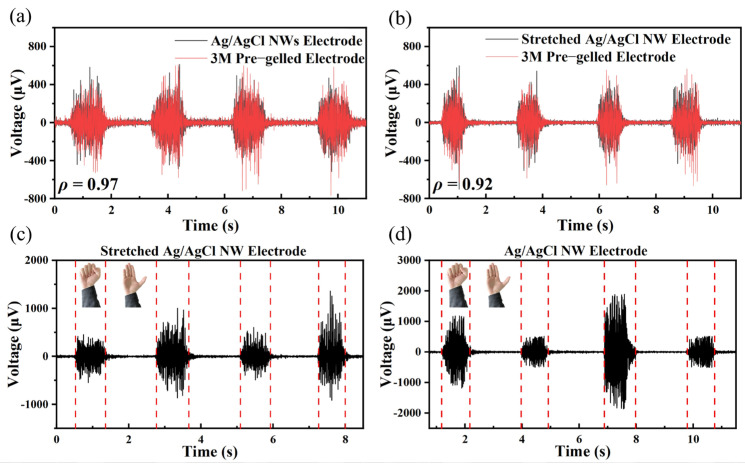
EMG signals measured for each electrode: (**a**) Ag/AgCl NW dry electrode and commercial wet electrode, (**b**) Ag/AgCl NW dry electrode with 50% tensile strain and commercial wet electrode, (**c**) EMG signals measured for Ag/AgCl NW dry electrode with different grip strengths, and (**d**) Ag/AgCl NW dry electrode after 150% deformation by stretching with different grip strengths. Note that the mutant signal between the two red dashed lines is the EMG during grip.

Figure 6a,b exhibit the EMG signals measured by the Ag/AgCl NW electrode without and with a 50% tensile strain, respectively, in reference to the signal given by the commercial wet electrode. The cross-correlation coefficients in Figure 6a and Figure 6b are 0.97 and 0.92, respectively, which demonstrate a strong correlation between the electrical signals obtained by the Ag/AgCl NW electrode (with or without stretching) and that obtained by the commercial wet electrode. Figure 6c,d display the EMG signals measured by the Ag/AgCl NW electrode and the stretched Ag/AgCl NW electrode, respectively, when the subjects clenched their fists with different levels of force. As the force increased, the amplitude of the electrical signal increased.

EMG signals generally have a high signal-to-noise ratio. The SNR of the signals measured by the commercial pre-gelled wet electrode, the Ag/AgCl NW electrode, and the stretched Ag/AgCl NW electrode were 25.6 dB, 28.9 dB, and 26.1 dB, respectively. In terms of the EMG signal measurements, the signals measured by our dry electrodes had a high SNR, which exceeded those of commercially available wet electrode measurement signals. The SNR of the measured signals from the stretched dry electrodes was reduced but still at a high level.

### 3.3. EEG Signal Measurement Results and Evaluation

**Figure 7:**
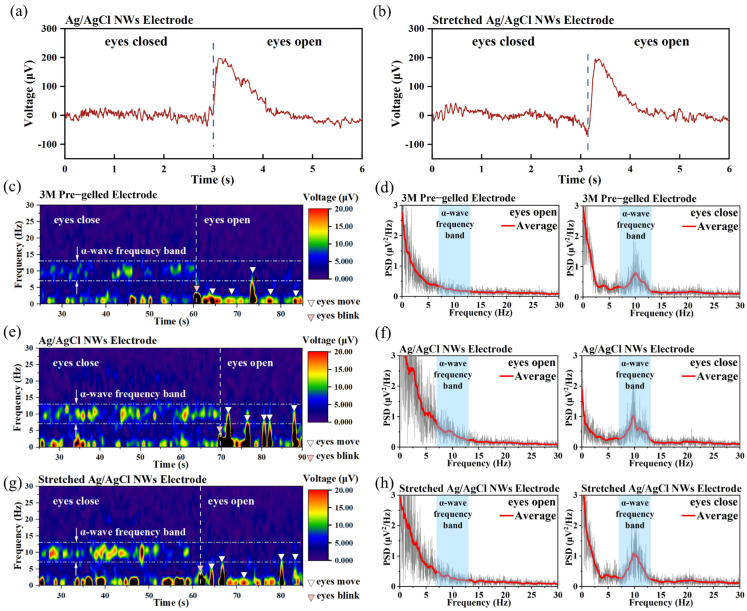
Raw EEG signals measured at each electrode: (**a**) Ag/AgCl NW dry electrode and (**b**) Ag/AgCl NW dry electrode with 50% tensile strain. Short-time Fourier transform (STFT, left) results of EEG signals measured at each electrode and fast Fourier transform (FFT, right) results after slicing the EEG signals after opening: (**c**,**d**) commercial wet electrode, (**e**,**f**) both Ag/AgCl NW dry electrodes, and (**g**,**h**) the Ag/AgCl NW dry electrode with 50% tensile strain. Note that the blue dashed lines in (**a**,**b**) distinguish the EEG signals after the eyes were opened.

Figure 7a,b show EEG signals measured using the Ag/AgCl NW electrodes, commercial pre-gelled wet electrodes, and stretched Ag/AgCl NW electrodes. A significant change in the EEG signal was observed before and after eye-opening. This suggests that the electrode has the ability to detect changes in EEG signals with a high sensitivity. However, further processing and analysis of the EEG signals is necessary for evaluating the signal quality. The SNR of EEG signals measured by electrodes is generally very low, which is the reason why most dry electrodes are unable to measure EEG. The SNR of the EEG signals measured by commercial wet electrodes, Ag/AgCl NW electrodes, and stretched Ag/AgCl NW electrodes were 6.45 dB, 6.6 dB, and 6.1 dB, respectively. In terms of the measurement of the EEG signals, the signals measured by our dry electrodes had high SNRs, which exceeded those of the commercial wet electrodes. The SNRs of the measurement signals of the stretched dry electrode had a lower SNR but were still at a high level. This indicates that the EEG signals measured by our flexible dry electrodes have high quality both before and after stretching.

The left side of other diagrams in Figure 7 shows the results of the short-time Fourier transform (STFT) when applied to raw EEG signals measured using commercial pre-gelled electrodes, Ag/AgCl NW electrodes, and stretched Ag/AgCl NW electrodes with 50% tensile strain. The right side of these figures shows the frequency domain results obtained by performing a fast Fourier transform (FFT) on the sliced signals before and after eye-opening. From Figure 7c,e,g, it can be seen that when the eyes are closed, significant voltage signals in the 8–13 Hz band can be observed using all three electrodes, indicating the presence of alpha waves. In the 8–13 Hz band, the signal amplitudes measured by the commercial wet electrode, Ag/AgCl NW electrode, and stretched Ag/AgCl NW electrode were 5–17 μV, 5–20 μV, and 5–23 μV, respectively. These signal amplitudes are consistent with those of α-waves measured clinically. When the eyes were open, significant voltage signals in the 8–13 Hz band were no longer observed, indicating the disappearance of α-waves. Moving the eyeball while the eyes were open produced large voltage signals in the low-frequency range, but there were no such signals when the subject was calm. These three electrodes can all significantly capture changes in the electrical signals.

Slicing the EEG signal before and after eye-opening and performing FFT on them separately can better demonstrate the presence and disappearance of α-waves, as shown in Figure 7d,f,h. During eye-opening, there was no significant energy in the 8–13 Hz frequency band for all three electrodes. However, during eye closure, significant energy was observed in the 8–13 Hz frequency band for all three electrodes, indicating that all three electrodes were capable of sensitively capturing the presence and disappearance of α-waves before and after eye-opening.

### 3.4. Quality Evaluation of Multiple Bioelectrical Signals

**Figure 8:**
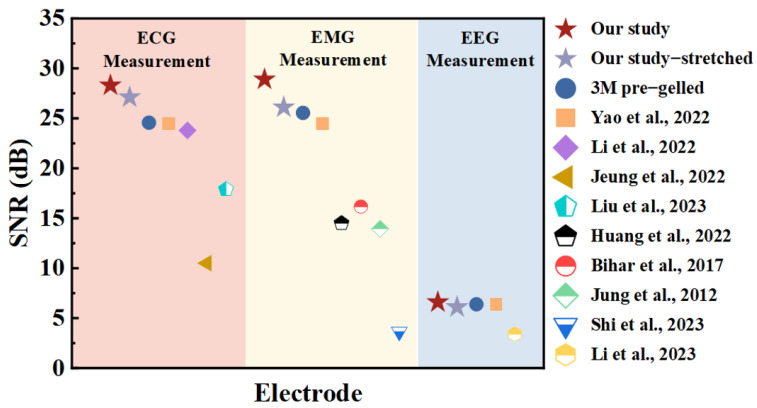
Comparison of signal-to-noise ratios of multiple bioelectric signals measured by different electrodes [4,12,30,31,32,33,34,35,36].

Some bioelectrical signals may not be effectively measured (e.g., EEG) if the signal-to-noise ratio of the signal measured by the electrode is low. And most dry electrodes have the problem of measuring signals with a low signal-to-noise ratio. High-quality dry electrodes can measure a wide range of bioelectric signals. The SNR provides a uniform measure of different types of bioelectric signals. Figure 8 illustrates the SNR of the signals measured by dry electrodes in previous studies measuring different bioelectric signals. The SNR of the signals measured by our Ag/AgCl NW dry electrodes for each of the bioelectrical signals measured are at the level of or greater than the SNRs of commercial pre-gelled electrodes. This illustrates that our dry electrode overcomes the low SNR of traditional dry electrodes.

There are several reasons why our Ag/AgCl NW dry electrodes achieve high SNR: first, the stretchability and adhesion of the PDMS substrate ensure a high fit between the electrodes and the skin, which results in a more stable signal quality and eliminates some of the motion artifacts. Secondly, the metal nanowire network has a lower impedance than conventional metal electrodes (e.g., disks) and provides better contact with the skin. Finally, and most importantly, the Ag/AgCl nanowire network prepared electrochemically prevents the oxidation of silver nanowires and increases the stability of the electrode. And the AgCl nanowire network has better electrical conductivity and, at the same time, can eliminate the polarization effect between the silver nanowire electrode and the skin, which makes Ag/AgCl NW dry electrodes more stable and reliable for the measurement of bioelectric signals, resulting in a higher SNR.

## 4. Conclusions

The flexible nanowire network dry electrode composed of Ag/AgCl nanowires (Ag/AgCl NW) and PDMS has been fabricated by electrochemical methods. The ECG, EMG, and EEG signals were measured using the Ag/AgCl NW electrode, the stretched Ag/AgCl NW electrode, and a mature commercial pre-gelled wet electrode. The quantitative analysis and evaluation of signal quality were performed based on waveform recognition, SQI parameters, and HRV parameters to explore the differences in measuring cardiac signals. All electrodes meet the SQI standards, and the Ag/AgCl NW and stretched Ag/AgCl NW electrodes display excellent signal quality. HRV analysis shows good agreement between the results measured by the dry (as well as the stretched) electrode with that of the wet electrode. The ECG signals are reliable, particularly in the 0.04 Hz to 0.4 Hz frequency band. The EMG signals measured by the Ag/AgCl NW electrodes are highly consistent with those measured by the commercial wet electrodes. The dry electrodes effectively capture the EEG signals with high sensitivity. Notably, our electrodes exhibit a significantly higher SNR compared to the commercial pre-gelled electrodes, which addresses the instability issue commonly associated with traditional dry electrodes. It is important to note that the bioelectric signals measured in this study were monitored over a short term (less than 24 h). In the future, in order to further extend our study to clinical use, a more detailed characterization of long-term monitoring and durability under different temperature and humidity fields is required. Even though the stretchable dry electrode offers a cost-effective, user-friendly alternative for various biomedical signal measurements, its flexibility and accuracy make it promising for long-term health monitoring in clinical settings, offering reliable and comfortable patient monitoring.

## Figures and Tables

**Table 1:** SQI parameters and signal quality of ECG signals measured by different electrodes.

Electrode	kSQI (Larger than 5)	pSQI (In [0.5, 0.8])	basSQI (Close to 1)	Quality
3M pre-gelled	7.33	0.67	0.99	Excellent
Ag/AgCl NW	9.55	0.71	0.99	Excellent
Stretched Ag/AgCl NW	8.85	0.70	0.99	Excellent

## Data Availability

The data that support the findings of this study are available from the corresponding author upon reasonable request.

## References

[1] Teng X., Zhang Y., Poon C.C.Y., Bonato P. (2008). Wearable Medical Systems for P-Health. IEEE Rev. Biomed. Eng..

[2] Luz E.J.D.S., Schwartz W.R., Cámara-Chávez G., Menotti D. (2016). ECG-Based Heartbeat Classification for Arrhythmia Detection: A Survey. Comput. Methods Programs Biomed..

[3] Zhang J., Wang B., Zhang C., Xiao Y., Wang M.Y. (2019). An EEG/EMG/EOG-Based Multimodal Human-Machine Interface to Real-Time Control of a Soft Robot Hand. Front. Neurorobot..

[4] Yao S., Zhou W., Hinson R., Dong P., Wu S., Ives J., Hu X., Huang H., Zhu Y. (2022). Ultrasoft Porous 3D Conductive Dry Electrodes for Electrophysiological Sensing and Myoelectric Control. Adv Mater. Technol..

[5] Jeong J.-W., Yeo W.-H., Akhtar A., Norton J.J.S., Kwack Y.-J., Li S., Jung S.-Y., Su Y., Lee W., Xia J. (2013). Materials and Optimized Designs for Human-Machine Interfaces via Epidermal Electronics. Adv. Mater..

[6] Sitaram R., Ros T., Stoeckel L., Haller S., Scharnowski F., Lewis-Peacock J., Weiskopf N., Blefari M.L., Rana M., Oblak E. (2017). Closed-Loop Brain Training: The Science of Neurofeedback. Nat. Rev. Neurosci..

[7] Araki T., Yoshimoto S., Uemura T., Miyazaki A., Kurihira N., Kasai Y., Harada Y., Nezu T., Iida H., Sandbrook J. (2022). Skin-like Transparent Sensor Sheet for Remote Healthcare Using Electroencephalography and Photoplethysmography. Adv Mater. Technol..

[8] Huang Z., Hao Y., Li Y., Hu H., Wang C., Nomoto A., Pan T., Gu Y., Chen Y., Zhang T. (2018). Three-Dimensional Integrated Stretchable Electronics. Nat. Electron..

[9] Patel S., Park H., Bonato P., Chan L., Rodgers M. (2012). A Review of Wearable Sensors and Systems with Application in Rehabilitation. J. NeuroEng. Rehabil..

[10] Kim H., Kim E., Choi C., Yeo W.-H. (2022). Advances in Soft and Dry Electrodes for Wearable Health Monitoring Devices. Micromachines.

[11] Yu Y.-H., Lu S.-W., Liao L.-D., Lin C.-T. (2014). Design, Fabrication, and Experimental Validation of Novel Flexible Silicon-Based Dry Sensors for Electroencephalography Signal Measurements. IEEE J. Transl. Eng. Health Med..

[12] Li J., Wang Y., Li C., Xu Z., Zhao Z., Raza S.A., Wang Y. (2022). Non-Contact Electrocardiogram Measuring Method Based on Capacitance Coupling Electrodes with Ultra-High Input Impedance. Rev. Sci. Instrum..

[13] Yokus M.A., Jur J.S. (2016). Fabric-Based Wearable Dry Electrodes for Body Surface Biopotential Recording. IEEE Trans. Biomed. Eng..

[14] Chi Y.M., Jung T.-P., Cauwenberghs G. (2010). Dry-Contact and Noncontact Biopotential Electrodes: Methodological Review. IEEE Rev. Biomed. Eng..

[15] Fayyaz Shahandashti P., Pourkheyrollah H., Jahanshahi A., Ghafoorifard H. (2019). Highly Conformable Stretchable Dry Electrodes Based on Inexpensive Flex Substrate for Long-Term Biopotential (EMG/ECG) Monitoring. Sens. Actuators A Phys..

[16] Wang L.-F., Liu J.-Q., Yang B., Yang C.-S. (2012). PDMS-Based Low Cost Flexible Dry Electrode for Long-Term EEG Measurement. IEEE Sens. J..

[17] Pater C. (2005). Methodological Considerations in the Design of Trials for Safety Assessment of New Drugs and Chemical Entities. Trials.

[18] Norton J.J.S., Lee D.S., Lee J.W., Lee W., Kwon O., Won P., Jung S.-Y., Cheng H., Jeong J.-W., Akce A. (2015). Soft, Curved Electrode Systems Capable of Integration on the Auricle as a Persistent Brain–Computer Interface. Proc. Natl. Acad. Sci. USA.

[19] Huang Y., Song Y., Gou L., Zou Y. (2021). A Novel Wearable Flexible Dry Electrode Based on Cowhide for ECG Measurement. Biosensors.

[20] Wang F., Li G., Chen J., Duan Y., Zhang D. (2016). Novel Semi-Dry Electrodes for Brain–Computer Interface Applications. J. Neural Eng..

[21] Masihi S., Panahi M., Maddipatla D., Hanson A.J., Fenech S., Bonek L., Sapoznik N., Fleming P.D., Bazuin B.J., Atashbar M.Z. (2022). Development of a Flexible Wireless ECG Monitoring Device with Dry Fabric Electrodes for Wearable Applications. IEEE Sens. J..

[22] Arquilla K., Devendorf L., Webb A.K., Anderson A.P. (2021). Detection of the Complete ECG Waveform with Woven Textile Electrodes. Biosensors.

[23] Wang R., Bai J., Zhu X., Li Z., Cheng L., Zhang G., Zhang W. (2022). A PDMS-Based Microneedle Array Electrode for Long-Term ECG Recording. Biomed. Microdevices.

[24] Qin Q., Li J., Yao S., Liu C., Huang H., Zhu Y. (2019). Electrocardiogram of a Silver Nanowire Based Dry Electrode: Quantitative Comparison with the Standard Ag/AgCl Gel Electrode. IEEE Access.

[25] Myers A., Zhu Y. (2014). Soft Dry Electrodes for Electrocardiogram with Conductive Silver Nanowires. MRS Proc..

[26] Yan X., Li B., Song K., Yang F., Wang Y., Wang C., Chi Y., Yang X. (2022). Ultra-Thin Foldable Transparent Electrodes Composed of Stacked Silver Nanowires Embedded in Polydimethylsiloxane. Mater. Res. Express.

[27] Abu-Saude M., Morshed B. (2018). Characterization of a Novel Polypyrrole (PPy) Conductive Polymer Coated Patterned Vertical CNT (pvCNT) Dry ECG Electrode. Chemosensors.

[28] Shih M., Kuo C.-T., Lin M.-H., Chuang Y.-J., Chen H., Yew T.-R. (2020). A 3D-CNT Micro-Electrode Array for Zebrafish ECG Study Including Directionality Measurement and Drug Test. Biocybern. Biomed. Eng..

[29] Dong P., Song Y., Yu S., Zhang Z., Mallipattu S.K., Djurić P.M., Yao S. (2023). Electromyogram-Based Lip-Reading via Unobtrusive Dry Electrodes and Machine Learning Methods. Small.

[30] Huang H., Wu N., Liu H., Dong Y., Han L., Wan S., Dou G., Sun L. (2022). Directional Sweat Transport and Breathable Sandwiched Electrodes for Electrocardiogram Monitoring System. Adv. Mater. Inter.

[31] Jeung J., Yun I., Kim Y., Seong S., Chung Y. (2022). Hierarchically Structured Flexible Electrode on Polyimide for Highly Sensitive and Reliable Biosignal Acquisition. IEEE Access.

[32] Bihar E., Roberts T., Saadaoui M., Hervé T., De Graaf J.B., Malliaras G.G. (2017). Inkjet-Printed PEDOT:PSS Electrodes on Paper for Electrocardiography. Adv. Healthc. Mater..

[33] Jung H., Moon J., Baek D., Lee J., Choi Y., Hong J., Lee S. (2012). CNT/PDMS Composite Flexible Dry Electrodesfor Long-Term ECG Monitoring. IEEE Trans. Biomed. Eng..

[34] Shi Z., Jiang B., Liang S., Zhang J., Suo D., Wu J., Chen D., Pei G., Yan T. (2023). Claw-Shaped Flexible and Low-Impedance Conductive Polymer Electrodes for EEG Recordings: Anemone Dry Electrode. Sci. China Technol. Sci..

[35] Li P., Meng Y., Li M., Xuan X., Xu S., Li H. (2023). An Electroencephalography Electrode Based on a Few-Layer Graphene/TiO2 Nanotube Nanoarchitecture for Application in Robot Arm Control. Sens. Actuators A Phys..

[36] Liu J., Liu K., Pan X., Bi K., Zhou F., Lu P., Lei M. (2023). A Flexible Semidry Electrode for Long-Term, High-Quality Electrocardiogram Monitoring. Adv. Compos. Hybrid Mater..

[37] Jeong J., Kim M.K., Cheng H., Yeo W., Huang X., Liu Y., Zhang Y., Huang Y., Rogers J.A. (2014). Capacitive Epidermal Electronics for Electrically Safe, Long-Term Electrophysiological Measurements. Adv. Healthc. Mater..

[38] Cho C., Kang P., Taqieddin A., Jing Y., Yong K., Kim J.M., Haque M.F., Aluru N.R., Nam S. (2021). Strain-Resilient Electrical Functionality in Thin-Film Metal Electrodes Using Two-Dimensional Interlayers. Nat. Electron..

[39] Myers A.C., Huang H., Zhu Y. (2015). Wearable Silver Nanowire Dry Electrodes for Electrophysiological Sensing. RSC Adv..

[40] Cui Z., Han Y., Huang Q., Dong J., Zhu Y. (2018). Electrohydrodynamic Printing of Silver Nanowires for Flexible and Stretchable Electronics. Nanoscale.

[41] Wu S., Yao S., Liu Y., Hu X., Huang H.H., Zhu Y. (2020). Buckle-Delamination-Enabled Stretchable Silver Nanowire Conductors. ACS Appl. Mater. Interfaces.

[42] Chen Y. (2022). Silver Nanowire Flexible Transparent Electrode toward Commercialization. Sci. China Chem..

[43] Chen X., Xu G., Zeng G., Gu H., Chen H., Xu H., Yao H., Li Y., Hou J., Li Y. (2020). Realizing Ultrahigh Mechanical Flexibility and >15% Efficiency of Flexible Organic Solar Cells via a “Welding” Flexible Transparent Electrode. Adv. Mater..

[44] Zhou W., Yao S., Wang H., Du Q., Ma Y., Zhu Y. (2020). Gas-Permeable, Ultrathin, Stretchable Epidermal Electronics with Porous Electrodes. ACS Nano.

[45] Xu F., Zhu Y. (2012). Highly Conductive and Stretchable Silver Nanowire Conductors. Adv. Mater..

[46] Pasaribu S.P., Ginting M., Masmur I., Kaban J. (2020). Silver Chloride Nanoparticles Embedded in Self-Healing Hydrogels with Biocompatible and Antibacterial Properties. J. Mol. Liq..

[47] Jones R.S., Draheim R.R., Roldo M. (2018). Silver Nanowires: Synthesis, Antibacterial Activity and Biomedical Applications. Appl. Sci..

[48] Yu Y. (2024). Biocompatible, Robust, Waterproof and Breathable PDMS-Based PU Fibrous Membranes for Potential Application in Wound Dressing. Mater. Today Commun..

[49] Conover M.B. (2002). Understanding Electrocardiography.

[50] Takala E.-P., Toivonen R. (2013). Placement of Forearm Surface EMG Electrodes in the Assessment of Hand Loading in Manual Tasks. Ergonomics.

[51] Makowski D., Pham T., Lau Z.J., Brammer J.C., Lespinasse F., Pham H., Schölzel C., Chen S.H.A. (2021). NeuroKit2: A Python Toolbox for Neurophysiological Signal Processing. Behav. Res..

[52] Li Q., Rajagopalan C., Clifford G.D. (2014). A Machine Learning Approach to Multi-Level ECG Signal Quality Classification. Comput. Methods Programs Biomed..

[53] Elgendi M., Jonkman M., DeBoer F. (2010). Frequency Bands Effects on QRS Detection. Pan.

[54] Zhao Z., Zhang Y. (2018). SQI Quality Evaluation Mechanism of Single-Lead ECG Signal Based on Simple Heuristic Fusion and Fuzzy Comprehensive Evaluation. Front. Physiol..

[55] Li Q., Mark R.G., Clifford G.D. (2008). Robust Heart Rate Estimation from Multiple Asynchronous Noisy Sources Using Signal Quality Indices and a Kalman Filter. Physiol. Meas..

[56] Clifford G.D., Behar J., Li Q., Rezek I. (2012). Signal Quality Indices and Data Fusion for Determining Clinical Acceptability of Electrocardiograms. Physiol. Meas..

[57] Orphanidou C., Drobnjak I. (2017). Quality Assessment of Ambulatory ECG Using Wavelet Entropy of the HRV Signal. IEEE J. Biomed. Health Inform..

